# Land use reshapes soil cercozoan functional architecture through soil chemistry and fungal networks

**DOI:** 10.1093/ismeco/ycag181

**Published:** 2026-06-27

**Authors:** Yijia Tang, Budiman Minasny, Peipei Xue, Ho Jun Jang, Alex McBratney

**Affiliations:** Sydney Institute of Agriculture & School of Life and Environmental Sciences, The University of Sydney, NSW 2006, Australia; Sydney Institute of Agriculture & School of Life and Environmental Sciences, The University of Sydney, NSW 2006, Australia; Sydney Institute of Agriculture & School of Life and Environmental Sciences, The University of Sydney, NSW 2006, Australia; Sydney Institute of Agriculture & School of Life and Environmental Sciences, The University of Sydney, NSW 2006, Australia; Sydney Institute of Agriculture & School of Life and Environmental Sciences, The University of Sydney, NSW 2006, Australia

**Keywords:** soil protists, nutrition, morphology, locomotion, sizes, soil nutrients

## Abstract

Soil protists are key regulators of microbial turnover and nutrient fluxes, yet their functional organization across managed landscapes remains poorly resolved. Among them, Cercozoa constitute one of the most abundant and ecologically influential lineages in terrestrial ecosystems. However, we lack a mechanistic framework explaining how protist functional composition reorganizes under land-use change and how abiotic filtering and biotic interactions jointly shape these shifts. Here, we examined cercozoan functional trait structure across cropping, pasture and woodland soils in the lower Namoi Valley, New South Wales, Australia, using 18S rRNA gene amplicon sequencing integrated with a curated trait database. Across land uses, communities were dominated by small, gliding bacterivores and omnivores, indicating strong convergence toward highly motile microbial grazers. Cropping soils exhibited higher functional alpha diversity and stronger coupling between trait composition and soil physicochemical gradients than woodland soils, suggesting intensified environmental filtering under agricultural management. Functional diversity was positively associated with fungal diversity and sodium, and negatively associated with organic carbon and total nitrogen, highlighting coordinated responses to both microbial networks and soil chemistry. Several trait categories showed consistent associations with fungal diversity, indicating that biotic coupling contributes to niche structuring alongside abiotic constraints. Together, these results demonstrate that land use reorganizes soil protist functional architecture through interacting physicochemical and biotic drivers, rather than through wholesale biodiversity loss. Trait-based protist metrics therefore provide a mechanistic lens for understanding soil ecological reassembly and offer a promising avenue for integrating microbial eukaryotes into soil health assessment under global change.

## Introduction

Soil protists are among the most diverse groups of microbial eukaryotes and can be highly abundant in soils (often ~10 000–100 000 individuals per gram of soil), with estimated annual production rates that may exceed 100 kg ha^−1^ [1–3]. These organisms play vital ecological roles in terrestrial ecosystems, particularly in nutrient cycling, where they function primarily as consumers with diverse feeding habits (e.g. grazing on bacteria, fungi, and other microeukaryotes), and include parasitic lineages that can influence plant growth and health [4–6]. Their functional diversity positions them as key components of soil food webs, regulating microbial turnover and nutrient mineralization and thereby indirectly shaping plant performance [7]. Despite their ecological significance, soil protists have historically been less studied than bacterial, fungal, and archaeal communities, reflecting challenges in extracting and accurately classifying protistan DNA from soils, limitations of cultivation-based methods, and the complexity of morphology-based identification [1, 2, 6]. Advances in high-throughput sequencing techniques have greatly improved our understanding of soil protistan diversity and community composition, highlighting the ecologically important lineages such as Cercozoa [8–10]. As one of the most diverse and functionally heterogeneous protistan groups, Cercozoa includes bacterivorous, fungivorous, and omnivorous taxa with a wide range of morphological and locomotory strategies, and occupies central roles in soil food webs through close linkages with bacterial and fungal communities [11, 12]. Yet, despite growing taxonomic knowledge, the mechanisms underlying how anthropogenic land-use change influences cercozoan community structure and function remain insufficiently understood [8, 13, 14].

Land use intensification profoundly alters soil environments by shifting resource availability, increasing physical disturbance, and restructuring plant communities, thereby reshaping both abiotic conditions and biotic interactions [2, 15, 16]. Although soil protistan communities respond to these factors, including biotic interactions with bacteria and fungi, and abiotic variables such as pH and moisture content, most studies have documented taxonomic shifts along natural environmental gradients (e.g. landscape structure or lithology) or within single land-use contexts (e.g. fertilization in crops or compartments in forests), rather than through systematic comparisons across contrasting anthropogenic types such as croplands, pastures, and woodlands [14, 16–19]. For example, bacterivorous Cercozoa dominated in the grasslands, comprising up to 69% of the community compared to dune systems [20], and intensive land use management in grasslands has been shown to promote distinct cercozoan functional groups [9]. Other studies have reported pronounced differences in cercozoan feeding modes across rhizosphere, root, soil, and litter compartments, but these findings have largely been restricted to forest ecosystems [5]. As a result, these findings highlight a critical gap: the absence of integrated, trait-based analyses that span diverse land use regimes and disentangle abiotic versus biotic drivers [13, 19]. These results expose a critical gap in soil ecology: the absence of an integrated framework to explain how protist functional structure reorganizes across land-use systems and how abiotic constraints and biotic networks jointly govern these shifts.

To bridge this, a functional trait–based approach offers a mechanistic advance over purely taxonomic studies, as traits capture organismal responses to environmental filters and biotic interactions, providing insights beyond species identities [11]. For soil protists like Cercozoa, traits such as trophic mode, morphology, locomotion, and body size are particularly relevant, governing feeding dynamics, soil pore navigation, and disturbance resilience. However, studies applying this framework across anthropogenic land-use gradients, while accounting for both abiotic soil properties and biotic factors like fungal communities, are notably scarce.

Here, we address this knowledge gap by investigating cercozoan communities in cropping, pasture, and woodland soils of the lower Namoi Valley, New South Wales (NSW), Australia. Traits including nutrition modes (e.g. bacterivore, autotroph, omnivore, plant parasite), morphology traits (e.g. naked amoebae, naked flagellates, naked amoeboflagellates, organic testate forms, siliceous testate forms and endoparasite, following the free-living vs. endoparasitic scheme of Dumack et al. 2020) [11], and locomotion modes (e.g. gliding, free swimming, and non-motile forms) shape responses to land use and contributions to soil processes. We test the following hypotheses: (i) Land-use type is associated with shifts in cercozoan trait composition, with cropland favouring smaller, fast-moving, bacterivorous taxa, and woodland supporting larger, more morphologically complex taxa typical of more stable conditions. (ii) Functional trait composition is associated with fungal community diversity and abiotic soil properties. (iii) Trait-based metrics of cercozoan community structure differ among the three land-use types examined here (cropping, pasture, woodland) to a degree comparable to, or greater than, differences detected in overall taxonomic composition.

## Materials & methods

### Sampling and soil properties

The study was conducted in the lower Namoi Valley in NSW, Australia, covering ~1700 km^2^. The region has a mean annual precipitation of ~646 mm, and a mean temperature of ~23°C [21]. Dominant soil types include *Vertosol* (black, dark brown, and brown), *Tenosol* (dark brown), and *Chromosol* (red) according to Australian Soil Classification, or the WRB equivalent of *Vertisol, Cambisol,* and *Luvisol* [22, 23]. The landscape has been subject to intensive agricultural activities since the early 1800s, with cropping and grazing as the dominant land uses [24], while few national parks remain without human disturbances.

A total of 46 soil samples were collected during June and July 2021, comprising cropping (*n* = 11), pasture (*n* = 11), and woodland (*n* = 24) sites. Sampling during this period aligned with the critical growth phase of winter crops in NSW, allowing us to capture the soil conditions most strongly influenced by land-use management. At each site, three subsamples were collected from the vertices of an equilateral triangle with 1meter sides at a depth of 0–15 cm following removal of surface vegetation. Subsamples were homogenized to form a single composite sample per site. Samples were sealed in sterile, airtight containers, and immediately transported to the lab on ice. The soil samples were then divided into two sets: one for physiochemical analysis and one stored at −20°C for molecular analysis.

Measurements of soil physiochemical properties were performed on soils that had been air-dried, ground to <2 mm with a hammer mill and sieved. Soil texture (clay, silt, and sand content) was analysed using a hydrometer [25]. Soil pH and electrical conductivity (EC) were measured using a 1:5 soil-to-water ratio with a combination electrode [26]. Measurements of soil organic carbon (OC) and total nitrogen (TN) were made using the dry combustion technique [26]. Total magnesium (Mg), sodium (Na), phosphorus (P), and sulphur (S) were measured using inductively coupled plasma-optical emission spectroscopy following standard protocols [26]. Detailed information, including geographical coordinates and soil type on each sampling location, is listed in Supplementary Material Table S2.

### Molecular analysis

Soil DNA was extracted from 0.25 g of fresh soil using the DNeasy® Powersoil® Kit (QIAGEN, CA, USA) implemented by the Australian Metagen Laboratory. The MMSF (CTTTAAGTTTCAGCTTTGC) and MMSR (GGTGCCAGCAGCCGCGGTA) primer pair was selected [27], as it amplifies the 18S V4–V5 region across a broad range of microbial eukaryotes, enabling simultaneous retrieval of fungal and protistan sequences from the same library. The DNA amplification polymerase chain reaction (PCR) programs were conducted using the MyTaq™ Red DNA Polymerase system (Bioline, UK) under the following cycling parameters: an initial denaturation at 95°C for 3 min, followed by 25 cycles (95°C for 20 s, 60°C for 20 s, and 72°C for 45 s), and a final extension period of 5 min at 72°C. DNA concentration and integrity were assessed using a Nanodrop™ 2000 (Thermo Scientific, MA, USA) and agarose gel electrophoresis prior to sequencing. Amplicon libraries were prepared following standard Illumina protocols and sequenced on an Illumina MiSeq instrument (PE300) by the Australian Metagenomics Laboratory. Demultiplexed FASTQ files were returned for downstream bioinformatic processing.

Raw sequencing reads were processed following the DADA2 pipeline in R (version 4.3), including quality filtering, denoizing, chimera removal, and inference of amplicon sequence variants (ASVs) [28]. ASVs with <10 reads across all samples or found in fewer than two samples were excluded to minimize bias. The remaining ASVs were classified taxonomically for eukaryotes using the Protist Ribosomal Reference (PR2) database (version 5.0.0) [29]. Fungal communities were defined as ASVs classified as fungi. Protistan communities were obtained by excluding fungi, streptophytes, animals, and unclassified groups (NAs) [13, 18]. Cercozoa were identified based on subdivision level PR2 assignments and extracted for downstream analyses. Sequencing depth and sampling sufficiency for Cercozoa were evaluated using abundance-based rarefaction, coverage-based rarefaction and extrapolation of Hill numbers (q = 0) in the “iNEXT” package [30], applied to pooled cercozoan ASV abundance data within each land-use category. Sampling sufficiency was further assessed using sample-based accumulation curves (incidence-based richness accumulation across samples) implemented in the “vegan” package. These analyses were performed on the post-filtered ASV table used for downstream analyses and are presented in Supporting Information Fig. S1.

Functional traits were assigned to cercozoan ASVs using a reference database of Dumack et al. (2020) [11], which synthesizes ecological and morphological trait information for Cercozoa from the published literature. Traits were classified into four categories: nutrition (bacterivore, omnivore, eukaryvore, autotroph, plant parasite), morphology (naked amoebae, naked flagellates, naked amoeboflagellates, organic testate forms, siliceous testate forms, and endoparasites), locomotion (gliding, free swimming, non-motile), and body size (<10, 11–30, 31–50, and >51 μm). The morphology category follows the Dumack et al. framework: it first separates free-living from endoparasitic forms, with free-living taxa further distinguished by cell architecture and shell type. Trophic mode, including plant parasitism, is captured separately under the nutrition category. Trait assignment was conducted at the genus level: ASVs confidently resolved to genus were assigned the corresponding genus-level trait profile where a reliable annotation was available. ASVs lacking genus-level resolution or documented ecological information were retained in the taxonomic dataset but excluded from trait-based functional analyses. Per-ASV taxonomy, read counts, sequences, and trait assignments are provided in Supplementary Material Table S1.

### Data analysis

For overall protistan and fungal analyses, ASV tables, taxonomy classifications, and soil sample metadata were combined into *phyloseq* objects using the phyloseq package in R (R 4.3.0) [31]. For taxonomic composition and beta-diversity analyses, ASV count tables were transformed to relative abundances using total sum scaling (TSS) to account for differences in sequencing depth among samples. Bray–Curtis dissimilarities were calculated from TSS-normalized relative abundance data using the *distance* function in phyloseq. Community structure was visualized using principal coordinates analysis (PCoA) via the *ordinate* function. Differences among land-use categories were tested using permutational multivariate analysis of variance (PERMANOVA) implemented in *adonis2* in vegan, with 999 permutations. Homogeneity of multivariate dispersion among land-use groups was assessed using *betadisper* with permutation testing to evaluate whether PERMANOVA results could be influenced by unequal within-group dispersion. Non-metric Multidimensional Scaling analysis was additionally performed using the *metaMDS* function to evaluate stress values in the *vegan* package [32]. Alpha diversity (Shannon and Simpson indices) was calculated from ASV count data using *estimate_richness* in phyloseq. Differences among land-use categories were assessed using Kruskal–Wallis tests followed by pairwise Wilcoxon tests with Benjamini–Hochberg correction for multiple comparisons.

For functional cercozoan analyses, the trait assigned ASV table, functional taxonomy table, phylogenetic tree, and metadata were combined into a *microtable* object using the *microeco* package [33]. Alpha diversity was calculated using cal_alphadiv within microeco, and differences among land uses were tested using non-parametric Wilcoxon rank-sum tests implemented in *trans_alpha$cal_diff* (method = “wilcox”). Functional community composition based on Bray–Curtis dissimilarities was assessed using PCoA ordination via *trans_beta*.

Random Forest analyses were used to evaluate the relative importance of abiotic and biotic predictors of cercozoan functional diversity using the *rfPermute* package [34]. For each functional response variable, predictor variables included fungal Shannon diversity, soil chemical properties (pH, organic carbon, total N, P, S, Na, and Mg), and soil physical properties (clay and sand content). Models were run with 500 trees and 100 permutation replicates using default mtry settings. Model performance was assessed using the out-of-bag error estimate. Predictor importance was quantified as the percentage increase in mean squared error, and statistical significance was determined using permutation-derived *P*-values [34]. Significant relationships were further examined using linear regression analysis via the lm function in the “stats” package [35]. All figures were generated in R (v4.3.0) using the packages *ggplot2, phyloseq*, and *microeco*, with figure assembly performed using *ggpubr* and *cowplot*.

## Results

### Structures of overall soil fungi and protists

After quality filtering, 1 433 523 18S rRNA gene reads were obtained, of which 55 099 were assigned to Cercozoa. Cercozoan read counts per sample ranged from 11 to 6033 (median 891; mean 1198), with comparable sequencing depth among land uses (mean reads per sample: cropping 1452; pasture 1200; and woodland 1080). Rarefaction/extrapolation and accumulation analyses confirmed high sample completeness across all three land use categories, with diminishing returns observed for both additional sequencing and additional sampling, supporting the robustness of cross-land-use comparisons (Supporting Information Fig. S1).

**Figure 1:**
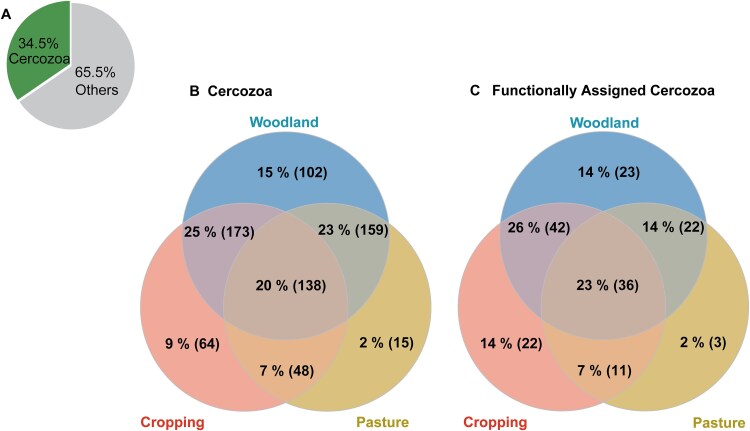
Distribution of cercozoan communities across land uses. (A) Proportion of Cercozoa in the overall protist community composition. (B) Venn diagram illustrating the distribution of overall cercozoan communities among cropping, pasture, and woodland; (C) Venn diagram showing the distribution of functionally assigned cercozoan traits across cropping, pasture and woodland.

Across all eukaryotic reads, fungi comprised 67.8% (567 ASVs) and protists 11.4% (699 ASVs) (Supporting Information Fig. S2). Ascomycota and Mucoromycota dominated the fungal communities, while Spirotrichea and Colpodea were the most abundant protistan classes (Supporting Information Fig. S3). Despite this dominance, Cercozoa represented a major protistan lineage overall, accounting for roughly one-third of total protistan ASVs and reads (Fig. 1A). Cercozoan ASVs showed both shared and land-use-specific distributions (Fig. 1B). Woodland contained the largest proportion of unique ASVs (15%), while pasture had the fewest (2%). A substantial fraction of ASVs was shared between land uses, particularly between woodland and the two managed land uses (pasture: 23%; cropping: 25%). When analyses were restricted to functionally assigned Cercozoa, the distribution shifted relative to overall Cercozoa (Fig. 1B), with a higher unique fraction in cropping (14%) and reduced overlap between woodland and pasture (14%) (Fig. 1C).

In the overall community analyses, alpha diversity of both fungal and protistan communities showed no significant differences among land uses (Supporting Information Fig. S4). Beta diversity differed significantly among land use types for both groups, with PERMANOVA R^2^ values of 0.118 (*P* < 0.001) for fungi and 0.078 (*P* < 0.001) for protists (Supporting Information Fig. S5). Tests for homogeneity of multivariate dispersion were also significant for both fungi (*F* = 3.825, *P* = 0.027) and protists (*F* = 8.099, *P* = 0.001).

**Figure 2:**
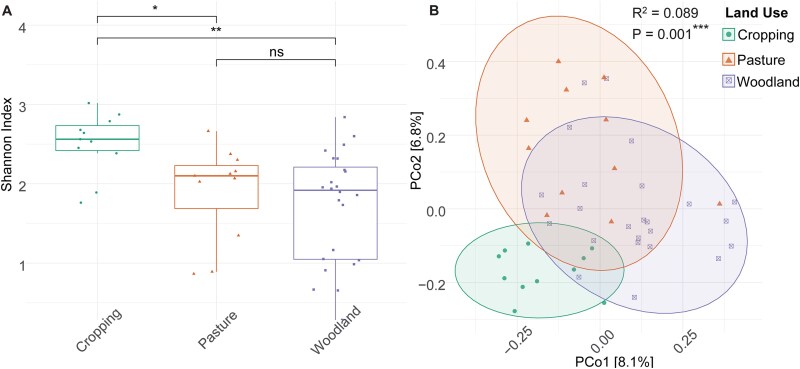
Alpha and beta diversity of overall functional cercozoan communities across different land uses. (A) Boxplot of Shannon diversity index showing the alpha diversity of functional cercozoan communities in cropping, pasture, and woodland. Significant differences between land uses are indicated by asterisks (^**^P < 0.01, ^*^P < 0.05), while "ns" denotes non-significant differences. (B) Principal Coordinates Analysis (PCoA) plot based on Bray–Curtis dissimilarity illustrating beta diversity of functional cercozoan communities across land uses.

In the functional Cercozoa dataset, Shannon diversity was highest in cropping and lowest in woodland (Fig. 2A). PCoA based on Bray–Curtis dissimilarities of trait-assigned Cercozoa showed significant separation among land uses (*R*^2^ = 0.089, *P* = 0.001) (Fig. 2B).

### Structure of functional cercozoan communities

**Figure 3:**
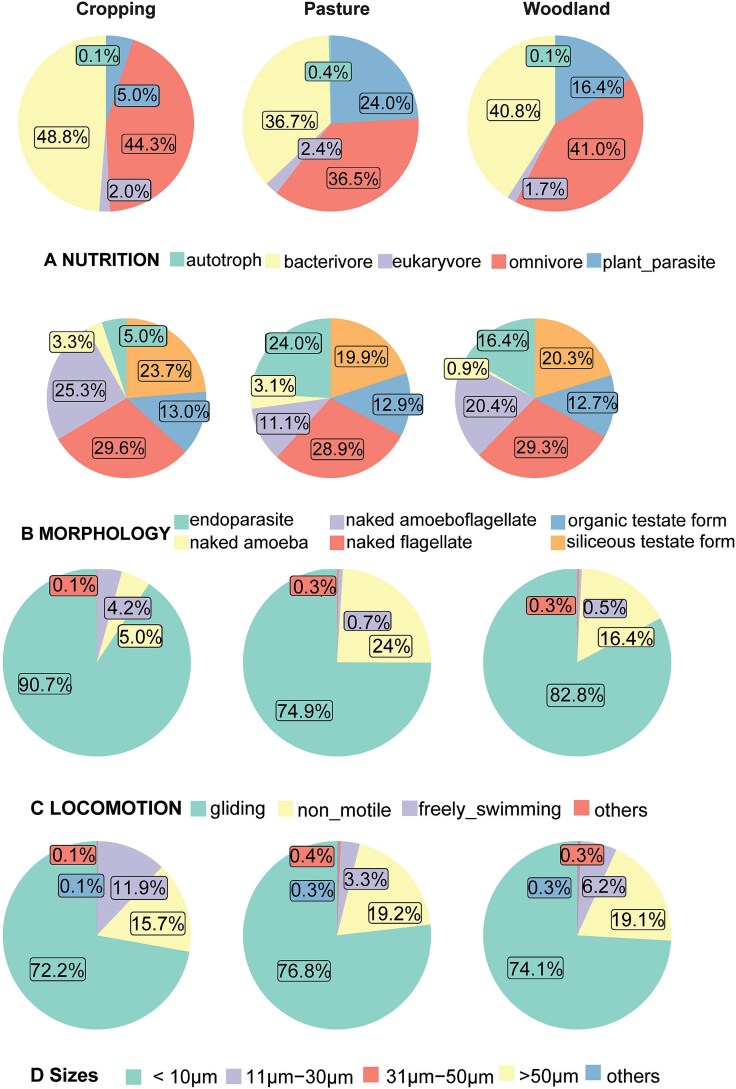
Pie charts showing the relative abundance of different functional cercozoan traits across four categories: (A) nutrition, (B) morphology, (C) locomotion and (D) sizes among cropping, pasture and woodland land uses.

**Figure 4:**
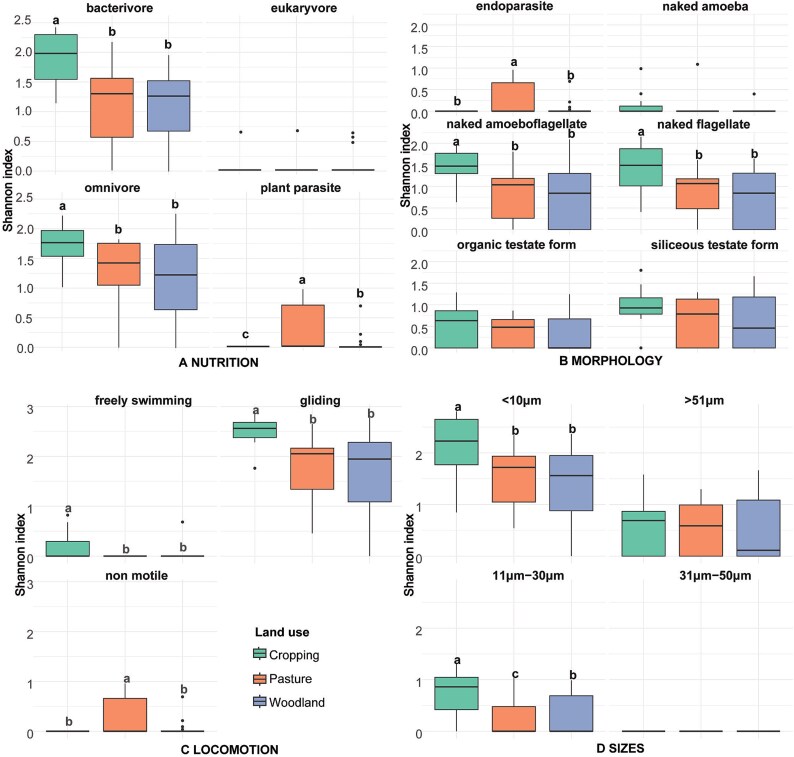
Alpha diversity of functional cercozoan traits across four categories: (A) nutrition, (B) morphology, (C) locomotion and (D) sizes among cropping, pasture and woodland land uses. Different letters indicate statistically significant differences (P < 0.05) within different land uses.

Functional trait information was assigned to 249 cercozoan ASVs, representing 35.6% of cercozoan ASVs and 33.6% of total cercozoan reads. The remaining 450 cercozoan ASVs lacked sufficiently resolved taxonomy or reliable trait information in the reference database and were therefore retained in the taxonomic analyses but excluded from trait-based functional analyses. Both the relative abundance and alpha diversity of functional cercozoan groups varied among land uses. Overall, cropping soils tended to support the greatest functional diversity, though the direction and magnitude of these differences depended on the trait category (Figs 3 and 4).

#### Nutrition

Bacterivores and omnivores dominated across all land uses, and were most abundant and diverse in cropping (each ~45%–50%; *P* < 0.001) (Figs 3A and 4A). These values declined in woodland and pasture (each ~36%–41%), with no significant difference in alpha diversity. Plant parasites showed the reverse pattern, being rare in cropping (~5%) but substantially more prevalent in pasture (~24%) (*P* < 0.05).

#### Morphology

Naked flagellates and naked amoeboflagellates were the predominant morphological groups, with the highest representation and diversity in cropping (each ~30%, *P* < 0.001) (Figs 3B and 4B). Endoparasites displayed the opposite trend, peaking in pasture (~24%) and remaining lowest in cropping (~5%) (*P* < 0.05). Other morphological groups varied weakly among land uses.

#### Locomotion

Gliding taxa overwhelmingly dominated across all land uses, reaching their highest relative abundance and diversity in cropping (~91%) (*P* < 0.01), and declining through woodland (~83%) and pasture (~75%) (Figs 3C and 4C). Non-motile taxa followed the reverse trend, increasing from ~5% in cropping to ~24% in pasture (*P* < 0.05). Freely swimming taxa were consistently rare across land uses (< 5%).

#### Size

Communities were consistently dominated by small taxa across land uses (<10 μm; ~72%–77%), but cropping supported significantly greater diversity within this size class than woodland and pasture (*P* < 0.001) (Figs 3D and 4D). Mid-sized taxa (11–30 μm) were more prominent in cropping (~12%) than in woodland (~6%) or pasture (~3%) (*P* < 0.001), while large taxa (>51 μm) increased in relative abundance toward pasture, with an opposing trend in alpha diversity.

### Effect of land use and other variables

**Figure 5:**
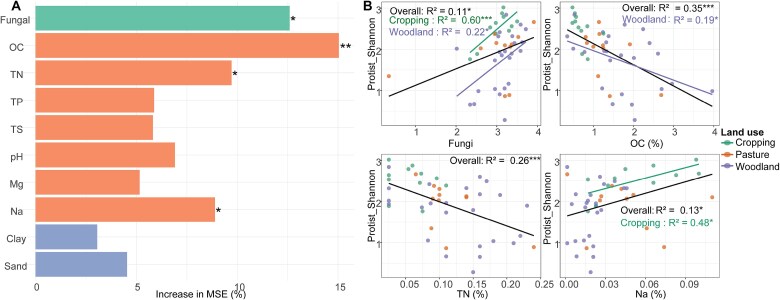
Significant factors influencing the alpha diversity of overall functional cercozoan communities. (A) Random forest models showing the effects of fungal alpha diversity, soil chemical, and physical factors on the alpha diversity of overall functional cercozoan traits, evaluated using mean squared error (MSE%) with the classification and regression tree (CART) methodology. Asterisks indicate statistically significant effects (^***^P < 0.001, ^**^P < 0.01, ^*^P < 0.05); (B) relationships between the alpha diversity of overall functional cercozoan communities and key environmental factors (fungi, organic carbon (OC), total nitrogen (TN), and sodium (Na)) across different land uses: Cropping, pasture, and woodland.

**Figure 6:**
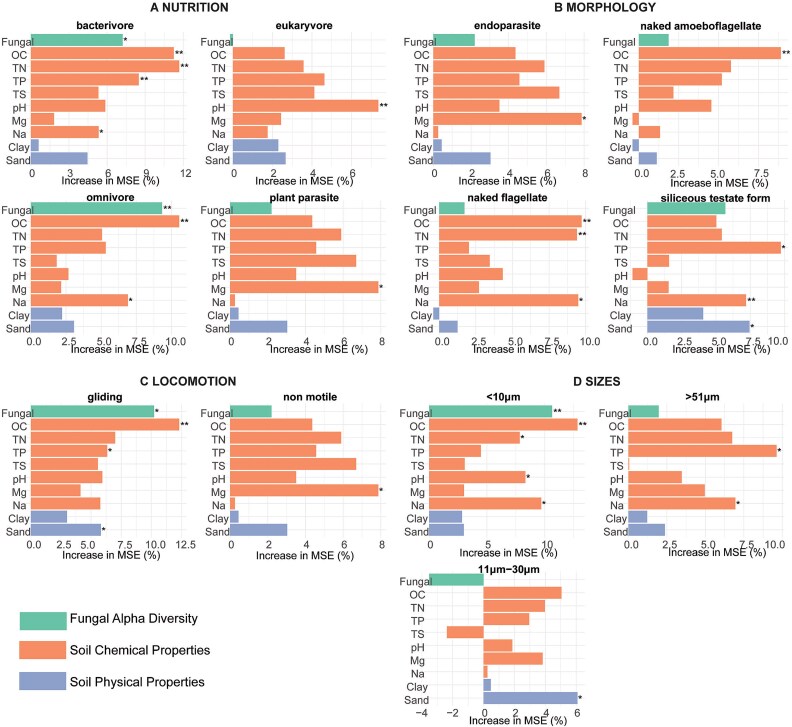
Random forest models illustrating the effects of fungal alpha diversity, soil chemical and physical factors on the alpha diversity of given functional cercozoan traits across four categories: (A) nutrition, (B) morphology, (C) locomotion and (D) sizes. The models are evaluated using the mean squared error (MSE%) with the classification and regression tree methodology. Asterisks indicate statistically significant effects (^***^P < 0.001, ^**^ P < 0.01, ^*^P < 0.05).

Random forest analyses identified fungal alpha diversity and soil properties (including OC, TN, TP, pH, Na, Mg, and sand content) as significant predictors of functional cercozoan diversity (Figs 5 and 6). Linear regression analyses confirmed a positive association between fungal diversity and cercozoan diversity overall (*R*^2^ = 0.11, *P* < 0.05), a relationship that was particularly pronounced in cropping soils (*R*^2^ = 0.6, *P* < 0.001) (Fig. 5B). Similarly, soil Na concentration showed a weak positive correlation with cercozoan diversity overall (*R*^2^ = 0.13, *P* < 0.05) but enhanced under cropping (*R*^2^ = 0.48, *P* < 0.05). OC and TN both exhibited negative overall correlations, with OC being especially strong in woodland soil (R^2^ = 0.19, *P* < 0.05).

When examined by trait category, the relative importance of predictors varied considerably (Fig. 6). For nutrition-related traits, OC emerged as the strongest and most consistent driver, showing negative associations with bacterivore and omnivore diversity, whereas fungal diversity was positively associated with these groups, particularly in woodland and cropping soils (Fig. 6A, Supporting Information Fig. S6). Soil Na showed positive effects in cropping for both groups, while pH strongly promoted eukaryvores diversity in woodland.

Morphological traits reflected similar strong environmental filtering (Fig. 6B, Supporting Information Fig. S7). In cropping soils, OC negatively influenced naked flagellates and amoeboflagellates, whereas Mg emerged as the primary predictor of endoparasites. Siliceous testate taxa were influenced primarily by TP in woodland soils, and more broadly with Na and sand concentration. Locomotion-based traits followed analogous patterns. Gliding organisms were positively associated with fungal diversity in cropping and woodland soils, but were suppressed by high OC in woodland and by sandy soils in cropping areas (Fig. 6C, Supporting Information Fig. S8). In contrast, non-motile groups showed limited environmental responsiveness and were associated only with Mg availability.

Size-based traits exhibited the clearest land-use differentiation (Fig. 6D, Supporting Information Fig. S9). Small-bodied organisms (<10 μm) were consistently promoted by fungal diversity in both cropping and woodland soils. In cropping systems, however, this size class was also influenced by soil chemistry, with OC exerting negative effects, and pH and Na level showing strong positive associations. In contrast, larger taxa (>51 μm) were negatively associated with TP in woodland soils, while mid-sized taxa (11–30 μm) were influenced primarily by sand content.

## Discussion

This study assessed how cercozoan functional traits vary across contrasting land use systems and provides a direct evaluation of the three hypotheses proposed in the Introduction. Hypothesis 1 was supported: land-use type was associated with distinct cercozoan trait assemblages, with cropping soils characterized by higher representation of smaller, gliding, bacterivorous and omnivorous taxa, whereas pasture and woodland soils supported greater representation of larger-bodied, non-motile and parasitic taxa. Hypothesis 2 was also supported: cercozoan functional composition was associated with both the measured biotic (fungal diversity) driver, and abiotic properties (OC, TN, Na, and pH), although the relative importance of these predictors varied among trait categories. Hypothesis 3 was partially supported: trait-based metrics captured clearer land-use-associated patterns than overall protistan alpha diversity, but the relatively modest separation in beta diversity suggests that functional responses were driven mainly by shifts in relative trait abundances rather than wholesale community turnover. Given the observational design, these patterns should be interpreted as associations rather than causal mechanisms, but they identify ecological gradients that warrant targeted experimental investigation.

At the community level, cropping soils exhibited higher functional cercozoan alpha diversity than woodland soils, with pasture generally intermediate. This gradient aligns with previous observations in managed ecosystems [14, 36, 37]. The Venn diagram of functionally assigned Cercozoa shows substantial overlap among cropping, pasture, and woodland, alongside measurable land-use-specific fractions (Fig. 1C), suggesting that land use is associated with shifts in functional composition without complete turnover. Although beta diversity differed significantly among land uses (Fig. 2B), separation was modest, and communities showed substantial overlap, indicating partial rather than complete compositional differentiation.

Given previous evidence for limited dispersal constraints at broad spatial scales, these patterns are most consistent with environmental filtering acting on a widely distributed regional species pool. Under this framework, land use appears to modulate the relative representation of functional strategies rather than exclude entire trait groups. Accordingly, we focus on functional alpha diversity as a sensitive metric to examine how soil properties and biotic interactions are associated with shifts in cercozoan ecological roles.

### Higher alpha diversity in cropping soils

Across all functional categories, cropping soils supported higher cercozoan alpha diversity than woodland soils (Fig. 4). Bacterivores and omnivores were also most abundant and diverse in cropping soils (Figs 3A and 4A), consistent with evidence for high bacterial abundance in a similar study area [38, 39], and with established predator–prey linkages in soil microbial food webs [40–42]. Management practices typical of cropping systems (e.g. fertilizers and tillage) can enhance bacterial productivity, which may contribute to conditions favouring heterotrophic protists [43–45]. The dominance of bacterivores and omnivores, therefore, likely reflects intensified microbial turnover and resource flux in managed soils.

Autotrophic cercozoans were rare across all land uses, with the few reads assigned predominantly to Chlorarachnea, a photosynthetic protistan group primarily known from coastal marine environments [11, 46]. Therefore, their detection at low abundance in soil should be interpreted cautiously and may partly reflect the limited resolution of 18S V4-V5 taxonomic assignment for closely related lineages, misclassification, or rare occurrences in soils. Regardless of identity, the low overall abundance of autotrophic Cercozoa is consistent with the limited representation of photosynthetic protists in many terrestrial soil habitats [47, 48]. The comparatively lower representation of eukaryvores and autotrophs among trait-annotated ASVs in cropping soils is consistent with, but does not by itself demonstrate, a shift in trophic structure toward bacterivory/omnivory, since the unannotated fraction (~64% of cercozoan ASVs) may include further taxa from these trophic categories.

### Opposite trends in parasitic and non-motile traits

In contrast to most functional groups, plant parasites, endoparasites, and non-motile taxa were most prevalent in pasture soils and least represented in cropping soils (Figs 3 and 4). Non-motile traits showed complete overlap with endoparasites in both relative abundance and alpha diversity, suggesting that the non-motile taxa detected here were primarily parasitic lineages, dominated by Plasmodiophorida (*Polymyxa betae, Sorosphaera veronicae*, and *Spongospora nasturtii*). This co-occurrence is consistent with immobility being characteristic of host-dependent lifestyles, allowing them to rely on their hosts for dispersal and survival.

The higher parasite representation in pasture relative to woodland aligns with previous findings that grass-dominated systems can support greater diversity of plant-associated parasites than tree-dominated soils [49]. Lower representation in woodland soils may reflect reduced availability of suitable hosts or plant-associated materials, since the dominant cercozoan parasites detected here *(Polymyxa, Sorosphaera, and Spongospora)* are obligate phytomyxid biotrophs [50]. Potential biotic interactions with more diverse microbial communities may also contribute, but cannot be tested with the present data and should be treated as hypotheses for targeted follow-up work. The reduced representation of plant parasites and endoparasites in cropping soils may be associated with frequent tillage, fertilizer inputs, and physical disturbance, which can alter soil structure and host availability [14, 51]. Additional stressors in open cropping systems (e.g. greater surface exposure and associated ultraviolet/desiccation stress) could further constrain their persistence [52]. These patterns suggest that parasitic functional strategies are particularly sensitive to land-use-mediated changes in host availability and environmental stability.

### Fungal diversity and soil chemistry as co-varying biotic and abiotic correlates

Cropping soils showed the greatest number of significant associations between soil properties and functional cercozoan diversity, whereas pasture and woodland soils showed fewer or no significant associations. This pattern suggests that environmental filtering is more pronounced in intensively managed soils, where repeated disturbance, reduced organic matter inputs and altered nutrient cycling can generate stronger abiotic gradients [53, 54].

**Figure 7:**
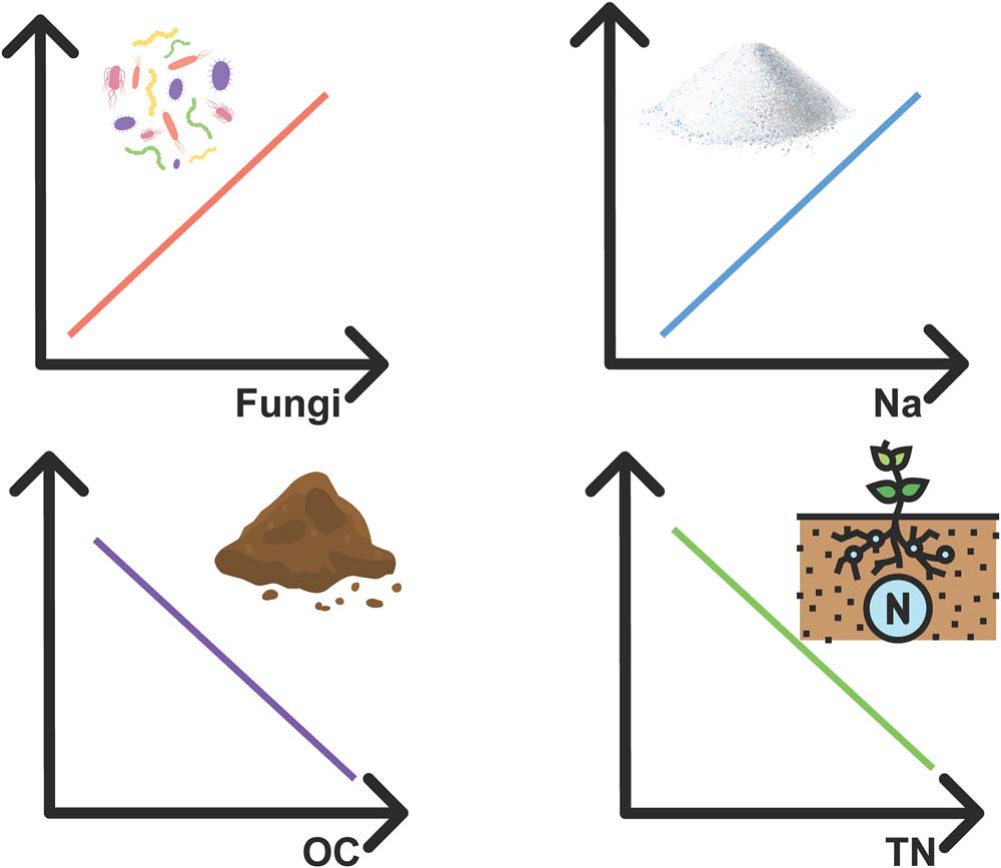
Schematic illustration showing consistent positive and negative correlations between major environmental factors (fungi, Na, OC and TN) and various cercozoan functional traits.

Across all samples, overall functional cercozoan diversity was positively associated with fungal diversity and Na content, and negatively associated with OC and total N (Fig. 5). These relationships are consistent with evidence that nutrient additions (particularly N fertilization) can reduce protist alpha diversity and alter functional composition in managed soils [55]. The positive association with Na likely reflects broader chemical context rather than a direct stimulatory effect, as salinity gradients are known to restructure protist communities and may co-vary with shifts in microbial prey composition and soil chemistry [56]. Consistently, multiple trait categories followed similar patterns (Figs 6 and 7), and alpha diversity of functional cercozoan traits was consistently higher in cropping than in pasture or woodland (Supporting Information Figs S6 – S9). Together, these patterns reinforce that land use-associated variation in soil physicochemical conditions co-varies with shifts in functional cercozoan diversity [13].

Fungal diversity emerged as a strong and consistent positive correlate of bacterivorous, omnivorous, gliding and small-bodied (<10 μm) traits. This supports the hypothesis that fungi contribute to habitat structuring and resource heterogeneity in soils, generating hyphal networks and associated bacterial assemblages that expand niche space for protists [57, 58]. Moreover, some taxa classified as bacterivorous may also be mycophagous [58], which could strengthen links between fungal and protistan diversity. Mycophagous protists can contribute to nutrient cycling and microbial turnover, highlighting the need for further work linking trait distributions to activity and process rates [58–60]. Dynamic fungal succession and carbon processing (e.g. cellulose decomposition) may also generate spatially heterogeneous resource patches that favour gliding and small-sized protists [57, 61].

Because soil properties differ systematically among land uses (Supplementary Information Table S3), some correlations may reflect land use contrasts rather than independent effects of individual variables. For example, woodland soils had higher OC (1.70%) and TN (0.13%) than cropping soils, which also exhibited lower functional cercozoan diversity; this contrast could contribute to negative associations between OC/TN and cercozoan diversity (Figs 5B and 7). Cropping soils also tended to accumulate higher Na. Rather than directly stimulating cercozoan metabolism, Na may act as a proxy for chemical filtering and associated shifts in microbial community composition, particularly as some free-living protists display tolerance to mild salinity [62, 63].

Finally, smaller-sized Cercozoa (< 10 μm) were positively associated with pH, particularly in cropping soils. This pattern may reflect variation in pH tolerance among protistan groups, with smaller taxa potentially favoured under neutral or slightly alkaline conditions due to surface-area-to-volume scaling and resource uptake efficiency [14, 64]. The fact that this association was most pronounced in cropping soils, where pH was consistently higher than in pasture or woodland, is consistent with reports that many protists, including smaller Cercozoa, perform well under neutral or weakly alkaline conditions [65–67].

## Conclusions

Overall, our results show that cercozoan communities are not merely passive responders to microbial prey. Land-use change reorganizes soil protist functional structure through coupled abiotic filtering and biotic interactions, rather than through simple biodiversity loss. Intensively managed systems exhibit stronger linkage between protist traits and soil chemistry, suggesting that disturbance amplifies environmental constraints on trophic organization. The consistent association between fungal diversity and key protist traits further highlights the role of biotic networks in structuring soil eukaryotic communities. These findings suggest that cercozoan functional traits have potential as a sensitive indicator of soil health. Trait-based protist metrics capture reorganization not visible in bulk chemistry, and functional protist diversity integrates signals of disturbance, nutrient status, and fungal network structure. Protists may serve as early-warning indicators of ecological reassembly under land-use change, but mechanistic confirmation will require targeted experiments and direct measurements of prey/host dynamics and protistan activity.

A limitation of this approach is that 18S sequencing provides lower fungal taxonomic resolution than ITS-based methods and yields fewer Cercozoa reads than lineage-targeted primers. Trait-based analyses were also restricted to the annotated Cercozoa subset, which may bias functional patterns toward better-characterized lineages. Bacterial communities, likely a primary food source for many Cercozoa, were not characterized here. The biotic correlates examined are therefore restricted to fungal diversity. Additional limitations include the spatial scope of sampling, the resolution of amplicon-based functional trait assignments, and the absence of direct measurements of protistan activity or prey dynamics. In conclusion, this study shows that land-use type reshapes cercozoan functional architecture along the cropping–pasture–woodland gradient. This restructuring is jointly associated with soil chemistry and fungal diversity, and trait-based metrics capture these shifts more clearly than overall protistan alpha diversity. These results position cercozoan functional traits as an informative complement to taxonomic surveys of soil protist communities. Future work integrating bacterial profiling, ITS-based fungal characterization, Cercozoa-targeted amplicon assays, and direct measurements of microbial activity across broader land-use and bioclimatic gradients would help clarify the causal pathways linking land use to protist functional ecology.

## Supplementary Material

Supplementary_material_ycag181

## Data Availability

Raw sequencing data have been deposited in the NCBI Sequence Read Archive under BioProject ID PRJNA1369739.
