# Supplementary material for: Land use reshapes soil cercozoan functional architecture through soil chemistry and fungal networks
Source: ISME Commun. 2026 Jun 27;6(1):ycag181. doi: 10.1093/ismeco/ycag181 (PMC13440301; doi:10.1093/ismeco/ycag181)
Supplement: Supplementary file 1 [file supplementary_material_ycag181.zip › Supporting Information_YJ Tang_ycag181.docx]

Supporting Information

Land use reshapes soil cercozoan functional architecture through soil chemistry and fungal networks

Yijia Tang^a^^*^, Budiman Minasny^a^, Alex McBratney^a^, Peipei Xue^a^, Ho Jun Jang^a^

^a^ School of Life and Environmental Sciences, The University of Sydney, Sydney, NSW 2015, Australia

***Corresponding author.**

Phone: +61 4 518 29180; Address: C81 - ATP - The Biomedical Building, The University of Sydney, Sydney New South Wales 2015, Australia; E-mail: yijia.tang@sydney.edu.au (Yijia Tang)

**Figures S1-S9**

**Fig. S1. Sequencing depth and sampling sufficiency of Cercozoa communities across land-use types.** (A) Coverage-based rarefaction curves showing sample completeness as a function of Cercozoa read numbers for cropping, pasture, and woodland soils. (B) Read-based rarefaction and extrapolation curves (Hill number q = 0) showing estimated Cercozoa ASV richness as a function of sequencing depth for each land-use category; solid lines indicate rarefaction and dashed lines indicate extrapolation beyond the observed read numbers. (C) Sample-based accumulation curve for Cercozoa ASV richness across all samples, showing diminishing gains in richness with increasing sample numbers. Shaded areas indicate ±1 standard deviation based on random permutations.

**Fig. S2.** Pie chart illustrating the proportions of fungal and protist communities based on 18S rRNA sequencing results.

**Fig. S3.** Relative abundance of fungal (A) and protistan (B) communities at the class level across cropping, pasture and woodland.

**Fig. S4.** Shannon and Simpson diversity indices of fungal (A, C) and protistan (B, D) communities across cropping, pasture and woodland.

**Fig. S5.** Non-metric multidimensional scaling (NMDS) plots showing the beta diversity of fungi (A) and protists (B) across cropping, pasture and woodland.

**Fig. S6.** Linear regression between the alpha diversity of different functional Cercozoan communities under Nutrition category and significant environmental factors across different land uses: cropping (green), pasture (orange), and woodland (blue).

**Fig. S7.** Linear regression between the alpha diversity of different functional Cercozoan communities under Morphology category and significant environmental factors across different land uses: cropping (green), pasture (orange), and woodland (blue).

**Fig. S8.** Linear regression between the alpha diversity of different functional Cercozoan communities under Locomotion category and significant environmental factors across different land uses: cropping (green), pasture (orange), and woodland (blue).

**Fig. S9.** Linear regression between the alpha diversity of different functional Cercozoan communities under Sizes category and significant environmental factors across different land uses: cropping (green), pasture (orange), and woodland (blue).
